# Dataset of Post-Event Survey of the 2024 Noto Peninsula Earthquake Tsunami in Japan

**DOI:** 10.1038/s41597-024-03619-z

**Published:** 2024-07-17

**Authors:** Masatoshi Yuhi, Shinya Umeda, Mamoru Arita, Junichi Ninomiya, Hideomi Gokon, Taro Arikawa, Toshitaka Baba, Fumihiko Imamura, Kenzou Kumagai, Shuichi Kure, Takuya Miyashita, Anawat Suppasri, Akio Kawai, Hisamichi Nobuoka, Tomoya Shibayama, Shunichi Koshimura, Nobuhito Mori

**Affiliations:** 1 School of Geosciences and Civil Engineering, Kanazawa University, Kanazawa, Ishikawa Japan; 2 Department of Civil and Environmental Engineering, Kanazawa Institute of Technology, Kanazawa, Ishikawa Japan; 3 Creative Society Design Research Area, Japan Advanced Institute of Science and Technology, Nomi, Ishikawa Japan; 4 Faculty of Science and Engineering, Chuo University, Bunkyo-Ku, Tokyo, Japan; 5 Graduate School for Social and Industrial Science and Technology, Tokushima University, Tokushima, Tokushima, Japan; 6 International Research Institute of Disaster Science, Tohoku University, Sendai, Miyagi Japan; 7 Pacific Consultants Co. Ltd., Osaka, Osaka, Japan; 8 Faculty of Engineering, Toyama Prefectural University, Imizu, Toyama, Japan; 9 Disaster Prevention Research Institute, Kyoto University, Uji, Kyoto, Japan; 10 Earthquake and Tsunami Observation Division, Seismology and Volcanology Department, Japan Meteorological Agency, Minato-Ku, Tokyo, Japan; 11 Graduate School of Science and Engineering, Ibaraki University, Mito, Ibaraki Japan; 12 Department of Civil and Environmental Engineering, Waseda University, Shinjuku-Ku, Tokyo, Japan; 13 School of Engineering and Applied Sciences, Swansea University, Swansea, UK

**Keywords:** Physical oceanography, Civil engineering

## Abstract

An earthquake with a moment magnitude of 7.5 (Mw) struck the northern Noto Peninsula, Ishikawa Prefecture, Japan, at 16:10 local time on January 1, 2024. This earthquake triggered a tsunami that propagated along the coastline of Ishikawa, Toyama, and Niigata Prefectures facing the Sea of Japan and significantly damaged coastal communities and infrastructure. Approximately 70 researchers from 23 universities or other institutes throughout Japan formed a joint research group to conduct a post-tsunami survey along a 340 km stretch of the coast. Based on the watermarks and traces of the tsunami, the inundation and run-up heights were surveyed using total stations, automatic optical levels, laser range finders, and a real-time kinematic (RTK) Global Navigation Satellite System (GNSS). The tidal correction was adjusted using astronomical tidal tables. In total, 303 survey records have been compiled, generating the NP2024TS (Noto Peninsula 2024 Tsunami Survey) dataset. This dataset provides comprehensive information on the inundation and run-up heights of the tsunami, which is useful for understanding tsunami characteristics and validating numerical tsunami models.

## Background & Summary

**Fig. 1:**
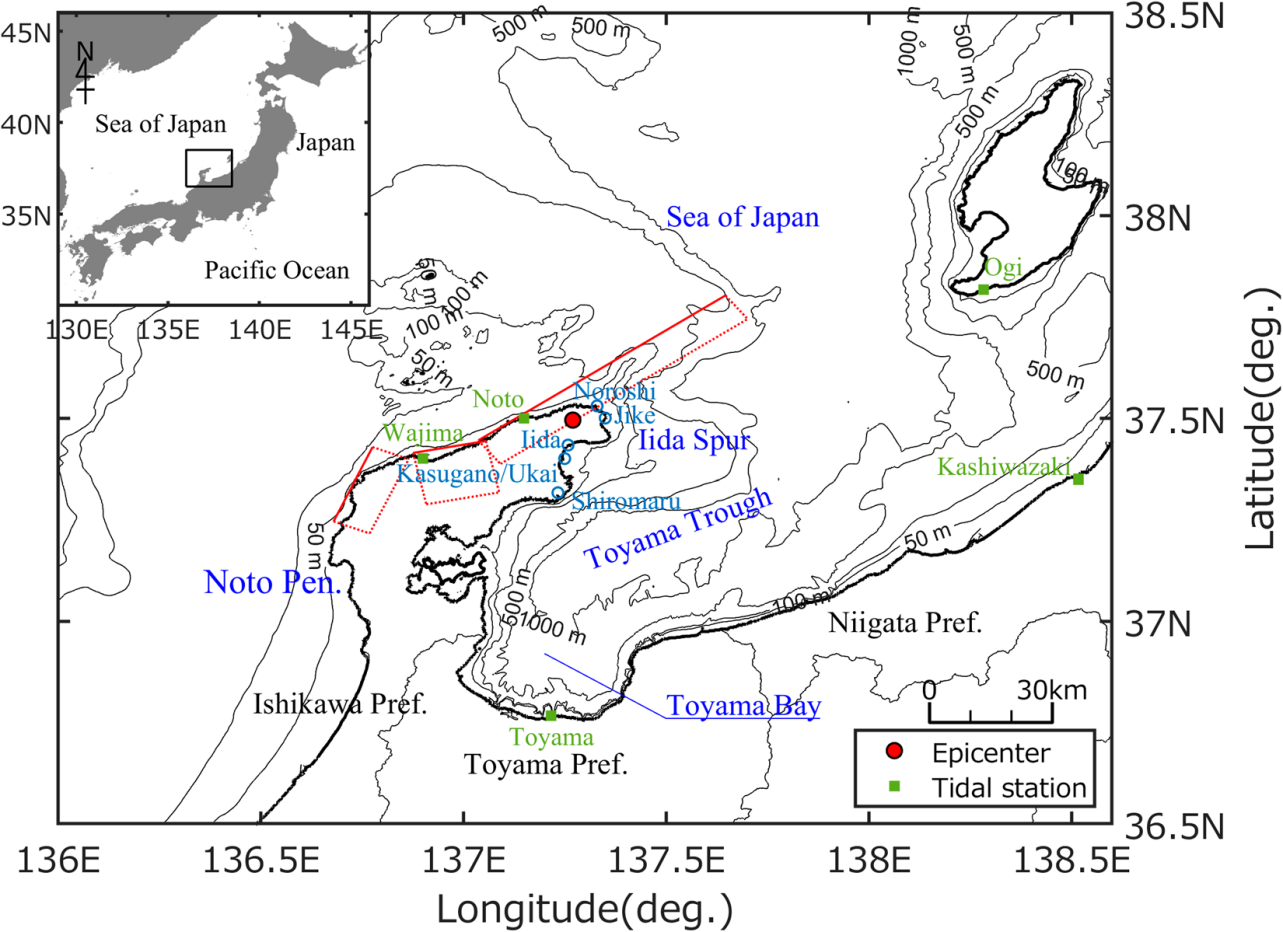
Site description. The red closed circle indicates the epicenter of the earthquake. The red rectangular boxes show the locations of the estimated earthquake source faults by GSI.

An earthquake with a moment magnitude 7.5 (Mw) struck the northern Noto Peninsula, Ishikawa Prefecture, Japan, on January 1, 2024, at 16:10 Japan Standard Time (+9 UTC) (Japan Meteorological Agency (JMA), https://www.jma.go.jp/jma/en/2024_Noto_Peninsula_Earthquake/index.html). According to the Geospatial Information Authority of Japan (https://www.gsi.go.jp/BOUSAI/20240101_noto_earthquake.html), the rupture of active faults extended over an area of more than 100 km from west to northeast of the peninsula (Fig. 1). This earthquake triggered a tsunami that significantly affected the surrounding coastline. Because the faults were located in the immediate vicinity of land, the tsunami hit the northern Noto Peninsula shortly after the earthquake. For example, the tsunami reached the Noroshi district of Suzu City within several minutes, and tsunami-induced inundation occurred approximately 30 minutes after the earthquake in the Iida, Kasugano, and Ukai districts of Suzu City, and Shiromaru district of Noto Town. The JMA issued a tsunami warning for Ishikawa, Toyama, and Niigata Prefectures and a tsunami advisory for the surrounding areas at 16:12. The tsunami warning for the Noto area was upgraded to major at 16:22. The tsunami subsequently struck the coastlines of Toyama and Niigata Prefectures.

Moreover, the tsunami propagated westward around the peninsula while significantly varying the propagation direction by refraction over the shallow area. At Shika Town in the western part of the peninsula, the highest tsunami elevation was observed approximately 1.5 hours after the earthquake. The tsunami eventually reached the Asian Continent on the opposite side of the Sea of Japan. On the Korean Peninsula, a tsunami was observed several hours after the earthquake. According to the Ministry of Land, Infrastructure, Transport, and Tourism, Japan (MLIT), the tsunami inundated over 1.90 × 10^6^ m^2^ of land in Ishikawa Prefecture and 4 × 10^4^ m^2^ in Niigata Prefecture (MLIT, https://www.mlit.go.jp/river/bousai/240101_noto/pdf/tsunamishinsui_higai_240202.pdf). As of February 20, 2024, the official number of fatalities attributed to the earthquake and tsunami was 241. The primary cause of death was the collapse of houses due to intense ground motion (JMA Seismic Intensity Scale 7). The number of tsunami victims was limited to two due to the rapid evacuation following the annual evacuation drills.

The complex bathymetry around the Noto Peninsula (Fig. 1) significantly affected tsunami propagation. Parts of the tsunami were significantly refracted over the Iida Spur (Fig. 1), while other parts propagated southward over the deep Toyama Trough (Fig. 1) with a depth of 1,200 m. The reflections from land and the diffraction/reflection around small islands further complicated tsunami behavior. The tsunami continued for a relatively long time, and the first wave was likely not the highest. Thus, the resulting distribution of inundation areas and patterns of related damage to coastal communities and infrastructure became spatially diverse. The most significant damage was concentrated in the northeastern part of Noto Peninsula, such as the Jike, Iida, Kasugano, and Ukai districts in Suzu City and the Shiromaru district in Noto Town. In contrast, inundation onto land was hardly observed in the northern and northwestern parts of the peninsula because the ground was significantly uplifted up to four meters, as reported by the Tectonic Geomorphology Research Group of the 2024 Noto Hanto Earthquake under the Association for Japanese Geographers (https://ajg-disaster.blogspot.com/2024/01/2024_2.html).

To effectively estimate future tsunami hazards not only in the Noto Peninsula but also in the coastal regions of high tsunami-risk countries worldwide, understanding the mechanism of tsunami generation, propagation, and inundation on land is necessary. Because tsunami behavior is sensitive to local underwater bathymetries, land topographies, and the locations of buildings, streets, and other elements of urban infrastructure, constructing a comprehensive on-site survey dataset is crucial for capturing and modeling tsunami generation, propagation, and inundation. For this purpose, approximately 70 researchers from more than 20 universities or other institutes throughout Japan formed a joint research group to implement a systematic post-tsunami on-site survey along a 340 km stretch of the coast. Watermarks and traces of tsunami inundation were identified across three prefectures from Ishikawa to Niigata. Based on high-quality field measurements conducted by the joint research group, an initial survey report describing the overall characteristics of tsunami inundation and the resulting damage was presented by Yuhi *et al*.^1^. In order to analyze tsunami inundation and the associated damage characteristics, on-site survey records of tsunami inundation and run-up heights were compiled into a comprehensive dataset.

This article summarizes the contents of the NP2024TS dataset and documents how the data, consisting of tsunami trace records, descriptive information, tidal corrections, and related photographs, were collected, processed, and compiled. This dataset is expected to be useful not only for clarifying the complex mechanism of the earthquake and tsunami but also for planning and implementing restoration and reconstruction in the affected areas. The dataset also provides an ideal benchmark for validating numerical tsunami models. Furthermore, we believe that this will also help in developing countermeasures against tsunamis and related complex disasters, which are a concern both domestically and internationally.

## Methods

### Data collection: conducting on-site field surveys

Tsunami surveys were conducted by a joint research group voluntarily formed by the Coastal Engineering Committee (CEC) of the Japan Society of Civil Engineers: The 2024 Noto Peninsula Tsunami Joint Survey Group (hereafter referred to as the survey group). This survey group comprised approximately 70 researchers and engineers with a wide range of expertise in tsunamis, coastal engineering/science, seismology, and geology. The survey group started a series of online meetings the day after the earthquake and began the preliminary survey within three days. The experience gained by a previous survey group in the CEC for the 2011 Great East Japan Earthquake^2,3^ was effectively utilized, especially in the early stages of activities, such as the coordination of different teams, methodology of sharing and updating information on the survey area, unified formatting of the survey records, and quality control of measurements. The priorities of the survey areas were determined based on preliminary information from aerial photographs (videos) and satellite images provided by government organizations. In addition, eyewitness accounts reported in various media were referred. Another important piece of information was the accessibility of roads to the survey area because most of the roads in the Noto Peninsula were severely damaged by the earthquake. Maps of available roads posted on the websites of the local government, automobile companies, and other academic associations were very useful. The individual teams shared updated information on overall progress and forthcoming survey plans through a mailing list. In cases where the planned areas of multiple teams overlapped, some areas were adjusted based on discussions. In this way, the survey group coordinated the activities of teams from more than 20 universities and research institutes.

On-site surveys were conducted according to the IOC-UNESCO guidelines for post-tsunami field survey^4^. The inundation and run-up heights were measured using total stations, automatic optical levels, laser range finders, and RTK/RTX (Real Time eXtended) GNSS systems. The inundation heights were determined from watermarks on the building walls and windows. The run-up height was determined from the maximum landward extent of debris and seawater marks. In order to maintain the reliability of the data, tsunami indicators were identified through discussions among multiple experts in individual survey teams based on their past experience and survey knowledge. The first survey was conducted on January 4, 2024. As of mid-February 2024, the total number of survey records was 303. The surveyed area covered an alongshore stretch of approximately 340 km, from Ishikawa Prefecture in the west to Niigata Prefecture in the east.

### Data processing: tidal corrections

**Fig. 2:**
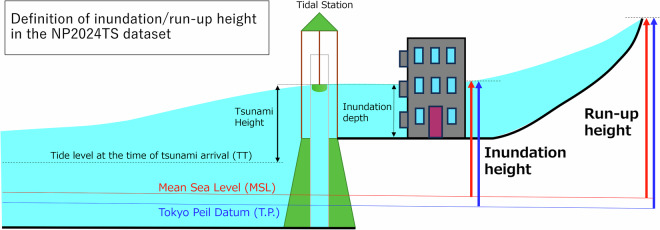
The definition of the inundation and run-up heights in the NP2024TS dataset.

In the NP2024TS dataset, the inundation and run-up heights were described as the elevation from the local mean sea level (MSL) (Fig. 2) because the arrival times of the highest tsunami were difficult to detect. The MSL for the five tidal stations in the survey area (Wajima, Noto, Toyama, Kashiwazaki, and Ogi; Fig. 1) was provided by the JMA for the period between 2018 and 2022 (https://www.data.jma.go.jp/kaiyou/db/tide/suisan/station.php). At all of these five stations, the sum of the amplitude of four principal tide components (M2, S2, K1, and O1) was less than 0.2 m (0.18 m according to JMA, https://www.data.jma.go.jp/kaiyou/db/tide/suisan/station.php). Accordingly, the difference between the tide level at the time of the highest tsunami arrival and MSL is considered within the typical tidal range of around ± 0.2 m at the site. Simultaneously, the dataset documents the inundation and run-up heights relative to the Tokyo Peil Datum (T.P.), the standard ground elevation in Japan based on the Tokyo Bay MSL (Fig. 2).

Generally, inundation surveys can be classified into two types: a method involving relative measurements from the sea surface to a watermark (total stations, automatic optical levels, and laser range finders) and a method that considers elevation measurements from a control point to a watermark (RTK/RTX-GNSS). Tidal corrections are required, regardless of the selection of the two methods, to prescribe the heights from both the local MSL and T.P. Tidal correction was conducted using an astronomical tidal table from the JMA as tide gauges along the Noto Peninsula (Wajima and Noto) could not be used because of the severe damage caused by the earthquake.

### Data compilation: Construction of tsunami trace dataset

After tidal correction, the survey results of the different teams were systematically collated and compiled. Subsequently, group members carefully examined the reliability and consistency of the dataset through online discussions. Finally, the inundation data were summarized in a unified Microsoft Excel format based on, but slightly revised from, that used in the 2011 Great East Japan Earthquake Tsunami Survey. Additional site descriptions were incorporated into the revised format for each survey record. Each record was systematically assigned a serial ID (#001 to #303) and a location ID consisting of the name of the municipality, district, and serial number within each district, such as SZ-Iida_01 for Suzu City, Iida District, and number 01. In the repository, site photographs related to each record were prepared in jpg format. The photographs were related to each record by name. Photographs corresponding to each record were named using the above IDs (e.g., #075_ SZ-Iida_01(a).jpg). Some records have multiple photos assigned. All individuals identifiable in the photographs consented to the open publication of their images.

**Fig. 3:**
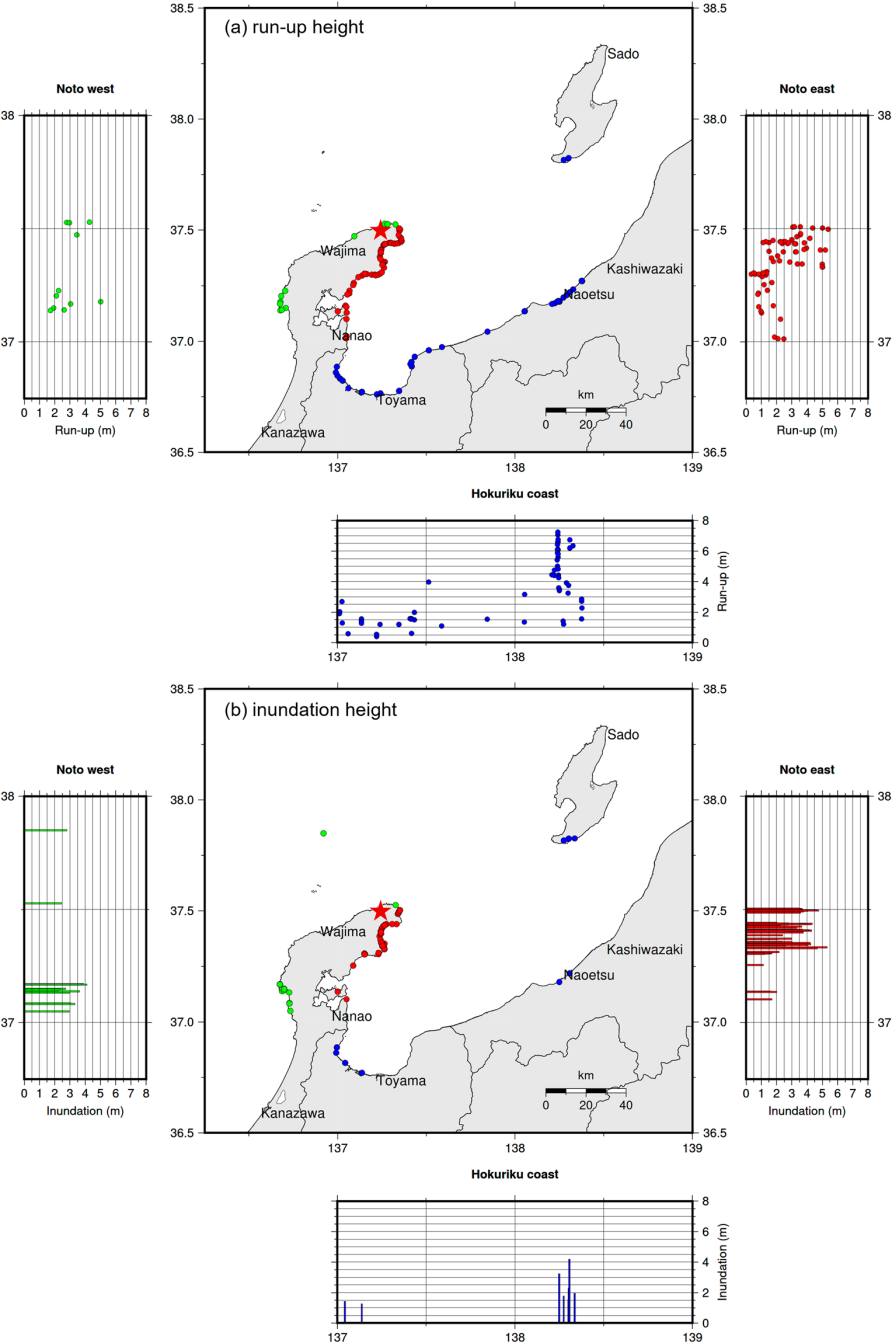
Distribution of the measured data over the whole area of the survey (MSL). (**a**) Run-up height, (**b**) Inundation height.

**Fig. 4:**
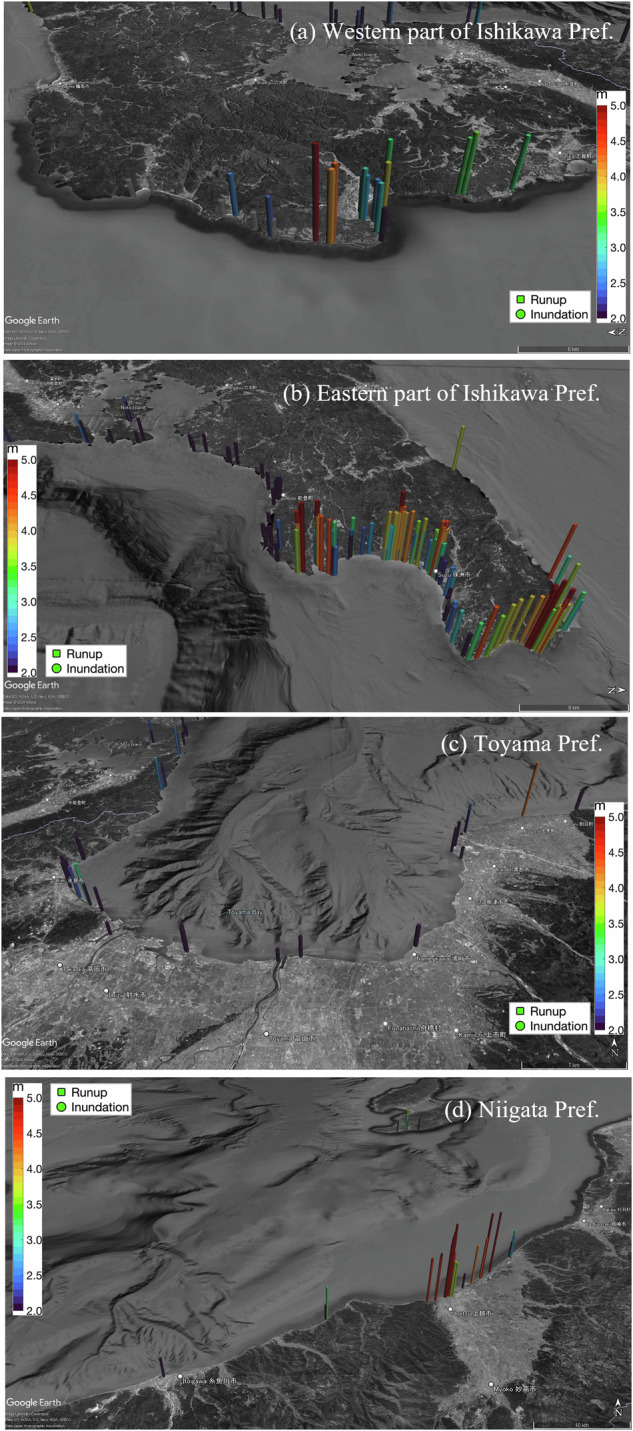
Regional run-up/inundation height (MSL) distribution around Ishikawa, Toyama, and Niigata Prefectures, Japan. The circular and square cylinders represent the inundation and run-up heights, respectively. (**a**) Western part of Ishikawa Prefecture, (**b**) Eastern part of Ishikawa Prefecture, (**c**) Toyama Prefecture, (**d**) Niigata Prefecture.

The spatial distributions of the inundation and run-up heights over the entire area are summarized in Fig. 3. High inundation and run-up heights were concentrated in the northeastern region of the Noto Peninsula. High run-up values were also observed in Niigata Prefecture. In contrast, the observed heights in the southeastern Noto Peninsula and Toyama Prefecture were low. A more detailed description of the characteristics of tsunami inundation based on the NP2024TS dataset and the associated damage was provided by Yuhi *et al*.^1^. The data were converted into Google Keyhole Markup Language (Fig. 4) and made available on the CEC website (https://coastal.jp/english/noto2024en/) along with supplementary information.

## Data Records

The full dataset in Microsoft Excel format comprising the survey results, descriptive information, inundation/run-up heights after tidal corrections, and supplementary photographs can be freely accessed through the figshare repository^5^.

The parameters described in the NP2024TS dataset are summarized in Table 1. The dataset is divided into four parts. The first part provides general information on the individual surveys. Subsequent to the record IDs, the location (latitude and longitude) of the identified tsunami indicator was shown in the coordinate frame of the World Geodetic System 84 (WGS84). The survey time and raw height measurements were also included. The type of tsunami height was classified into four categories (Table 2). The second part provides descriptive information on inundation/run-up height measurements, including the target tsunami trace, measurement method, equipment used for the survey (name and manufacturer), the influence of splash or wind/swell waves, and a description of the surrounding conditions. The reliability of the measurements was categorized into four levels (Table 3). The third and last parts provide information on the tidal correction and the inundation/run-up height after tidal correction, respectively. In addition, the NP2024TS dataset contained photographs associated with the identified tsunami indicators.

**Table 1:** List of parameters described in the NP2024TS dataset.

	Parameter	Unit
General information on tsunami trace measurement	Serial ID	—
	Team ID (for data management purposes)	
	Location ID	—
	Latitude	degree/minute/second
	Longitude	degree/minute/second
	Date of survey	month/date/year
	Time of survey (Local time; UTC + 9)	hour/minute
	Raw measurement of inundation/run-up height	m
	Classification of tsunami height	—
	Distance from shoreline (optional)	m
Descriptive information on the situation of the survey	Target tsunami trace	—
	Measurement method	—
	Equipment used for the survey	—
	Influence of splash or wind/swell waves	—
	Description of surrounding conditions	—
	Reliability level	—
Information on tidal correction	Tide level at the time of the survey	m
	Data source location of tidal correction	—
Inundation/run-up height after tidal correction	Inundation/run-up height with respect to Tokyo Peil datum	m
	Inundation/run-up height above mean sea level	m

**Table 2:** Classification of tsunami height.

R	Run-up height
I	Inundation height
P	The height of tsunami trace in a port (no inundation)
W	Run-up height which is difficult to distinguish from that by wind waves or swells

**Table 3:** Reliability level.

A Highest reliability	Clear watermark on a wall (or window) or drifted object that was unaffected by wind and measured by RTK/RTX-GNSS, total station, and other professional surveying instruments
B High reliability	1) Clear watermark on a wall (or window) or drifted object that was unaffected by wind and measured by hand-held laser range finder/hand level
	2) Drifted object possibly influenced by wind measured by RTK/RTX-GNSS, total station, and other professional surveying instruments
C Moderate reliability	1) Drifted object that was possibly influenced by wind and measured by hand-held laser range finder/hand level
	2) Clear watermark on a wall (or window) or drifted object that was measured by the combined use of staff and digital ground information data
	3) Tsunami traces based on eyewitness testimony measured by RTK/RTX-GNSS, total station, and other professional surveying instruments, laser range finders/hand levels
D Weak reliability	Tsunami traces based on eyewitness testimony measured by the combined use of staff and digital ground information data

## Technical Validation

The on-site surveys referred to the IOC-UNESCO guideline^4^ for post-tsunami field surveys. Each survey was conducted by a small team of experts. At each site, the reliability of the watermark or trace was carefully verified and cross-checked among the members. Careful inspections were conducted in light of the surrounding conditions (e.g., damage to the house). When different teams conducted their measurements over a close vicinity (e.g., tsunami traces placed on identical buildings/houses), an intrasite comparison was conducted to maintain consistency within the entire dataset. Occasionally, locally high inundation/run-up heights have been reported compared with the surrounding situation. In such cases, efforts were made to conduct additional surveys by another team at the same (nearby) location for comparison. In the dataset, descriptive information and related photographs were included to enable users to confirm the reliability of their observations. For example, when the observed trace was possibly influenced by wind, wind waves, swells, splash, or microtopography, this was noted in the descriptive information columns.

The vertical accuracy of the inundation and run-up heights measured by the total stations, other professional surveying instruments, and RTK/RTX-GNSS were considered to be within a few centimeters. A similar error range may appear when the sea surface was visually determined at the survey site if the wave-induced surface variation near the shoreline was not negligible.

The surveyed area facing the Sea of Japan is microtidal: the typical tidal range is around ±0.2 m. Thus, the error arising from the tidal correction process was considered to be within that range.

The distance to the shoreline measured by the equipment mentioned above was expected to be accurate within a few centimeters. The accuracy of the horizontal locations (longitude and latitude) of the traces and watermarks was expected to be similar when RTK/RTX-GNSS was used. However, it should be noted that the horizontal location of the tsunami indicators was often determined by handheld GNSS equipment (e.g., Garmin GPSMAP 66i, Garmin Oregon 750TJ) when the total stations and laser range finders were used; in such cases, the longitude and latitude description may include an error of several meters.

## Data Availability

No customized code was produced to prepare or analyze the dataset.

## References

[1] Yuhi, M. *et al*. Post-event survey of the 2024 Noto Peninsula earthquake tsunami in Japan. *Coastal Eng. J*., 1–14, 10.1080/21664250.2024.2368955 (2024). 10.1038/s41597-024-03619-z PMC11254902 39019890

[2] Mori N, Takahashi T, Yasuda T, Yanagisawa H (2011). Survey of 2011 Tohoku earthquake tsunami inundation and run-up. Geophys. Res. Lett..

[3] Mori N, Takahashi T (2012). and The 2011 Tohoku Earthquake Tsunami joint survey group. Nationwide post-event survey and analysis of the 2011 Tohoku Earthquake Tsunami. Coast. Eng. J..

[4] UNESCO International Tsunami Survey Team (ITST). Post-Tsunami Surve Field Guide, *IOC Manuals and Guides* 37, 2nd edition, Paris, UNESCO (2014).

[5] Yuhi M (2024). figshare.

